# Identification of a transitional fibroblast function in very early rheumatoid arthritis

**DOI:** 10.1136/annrheumdis-2017-211286

**Published:** 2017-08-28

**Authors:** Andrew Filer, Lewis S C Ward, Samuel Kemble, Christopher S Davies, Hafsa Munir, Rebekah Rogers, Karim Raza, Christopher Dominic Buckley, Gerard B Nash, Helen M McGettrick

**Affiliations:** 1 Rheumatology Research Group, Arthritis Research UK Centre of Excellence in the Pathogenesis of Rheumatoid Arthritis, Institute of Inflammation and Ageing, Birmingham, UK; 2 Department of Rheumatology, Sandwell and West Birmingham Hospitals NHS Trust, Birmingham, UK; 3 University Hospitals Birmingham NHS Foundation Trust, Birmingham, UK; 4 Institute of Cardiovascular Sciences, University of Birmingham, Birmingham, UK

**Keywords:** Fibroblasts, endothelial cells, lymphocytes, rheumatoid arthritis, adhesion

## Abstract

**Objectives:**

Synovial fibroblasts actively regulate the inflammatory infiltrate by communicating with neighbouring endothelial cells (EC). Surprisingly, little is known about how the development of rheumatoid arthritis (RA) alters these immunomodulatory properties. We examined the effects of phase of RA and disease outcome (resolving vs persistence) on fibroblast crosstalk with EC and regulation of lymphocyte recruitment.

**Methods:**

Fibroblasts were isolated from patients without synovitis, with resolving arthritis, very early RA (VeRA; symptom ≤12 weeks) and established RA undergoing joint replacement (JRep) surgery. Endothelial-fibroblast cocultures were formed on opposite sides of porous filters. Lymphocyte adhesion from flow, secretion of soluble mediators and interleukin 6 (IL-6) signalling were assessed.

**Results:**

Fibroblasts from non-inflamed and resolving arthritis were immunosuppressive, inhibiting lymphocyte recruitment to cytokine-treated endothelium. This effect was lost very early in the development of RA, such that fibroblasts no longer suppressed recruitment. Changes in IL-6 and transforming growth factor beta 1 (TGF-β_1_) signalling appeared critical for the loss of the immunosuppressive phenotype. In the absence of exogenous cytokines, JRep, but not VeRA, fibroblasts activated endothelium to support lymphocyte.

**Conclusions:**

In RA, fibroblasts undergo two distinct changes in function: first a loss of immunosuppressive responses early in disease development, followed by the later acquisition of a stimulatory phenotype. Fibroblasts exhibit a transitional functional phenotype during the first 3 months of symptoms that contributes to the accumulation of persistent infiltrates. Finally, the role of IL-6 and TGF-β_1_ changes from immunosuppressive in resolving arthritis to stimulatory very early in the development of RA. Early interventions targeting ‘pathogenic’ fibroblasts may be required in order to restore protective regulatory processes.

## Introduction

Fibroblasts are a type of mesenchymal stromal cell with immunomodulatory capabilities.^1^ They display distinct spatial identities^2 3^ that govern their behaviour and allow them to establish tissue-specific ‘address-codes’.^4^ It is these address codes that actively regulate the recruitment of leucocytes to inflamed sites and their subsequent behaviour.^1^ Fibroblasts achieve these effects in part by conversing with neighbouring vascular endothelial cells (EC) to regulate leucocyte adhesion.^1^ We have previously reported that dermal fibroblasts potently downregulate the responsiveness of EC to cytokines, suppressing lymphocyte recruitment in an interleukin 6 (IL-6) and transforming growth factor beta 1 (TGF-β_1_) dependent manner.^5^ Consequently, each inflammatory response is contextual, defined by the phenotype of the local fibroblast population.

In rheumatoid arthritis (RA), the stable reprogramming of synovial fibroblasts disrupts their protective regulatory processes, promoting their survival and enhancing their production of proinflammatory agents and proteases for example.^6^ Additionally, rheumatoid synovial fibroblasts invade human cartilage in an severe combined immunodeficiency (SCID) model of arthritis^7 8^ and appear to display tropism for damaged tissue, migrating to distant cell-free cartilage in vivo, potentially ‘spreading’ disease.^9^ This pathogenic phenotype causes RA fibroblasts to bypass many of the regulatory checkpoints that coordinate the successful resolution of an inflammatory episode. Indeed, we have shown that rheumatoid synovial fibroblasts activate endothelium to inappropriately recruit leucocytes,^5 10^ while simultaneously blocking leucocyte apoptosis.^11^ Thus, rheumatoid synovial fibroblasts are capable of generating and supporting persistent leucocyte infiltrates.

Fibroblasts are endogenous regulators of inflammation, and in our hands demonstrate a spectrum of responses, ranging from suppression of cytokine-induced responses to stimulation of a persistent leucocyte influx.^5^ This suggests that at some stage during the development and progression of RA,^12^ immunomodulatory capability is lost, and a proinflammatory phenotype is acquired in synovial fibroblasts. However, it remains unclear when these events occur. Here, we show for the first time that fibroblast–EC interactions evolve with disease progression and that fibroblasts at the earliest phase of RA exhibit a transitional functional phenotype that contributes to the accumulation of persistent infiltrates.

## Materials and methods

### Isolation of human fibroblasts, ECs and lymphocytes

Synovial tissue samples were obtained by ultrasound-guided biopsy^13^ from treatment-naive patients with a new onset of clinically apparent arthritis and a symptom duration of ≤12 weeks, who at follow-up had either a resolving arthritis (Res) or fulfilled RA classification criteria (very early RA; VeRA).^14^ Patients were classified as having resolving arthritis if there was no clinical evidence of synovial swelling at any peripheral joint (out of a swollen joint count of 66 joints) on final examination at least 1 year after initial presentation, in the absence of disease-modifying antirheumatic drugs (DMARD) or glucocorticoid therapy for at least the previous 3 months.^15^ In addition, synovial tissue samples were collected from subjects (A) with established, treated RA undergoing joint replacement (JRep) surgery; or (B) undergoing exploratory arthroscopy for unexplained joint pain with no macro or microscopic evidence of inflammation (non-inflamed—NI). RA was classified according to 2010 American College of Rheumatology criteria.^16^ Prior to biopsy, the extent of greyscale synovitis and power Doppler enhancement within the synovium of the biopsied joint was systematically graded using a 0–3 scale.^14^ Fibroblasts were isolated as previously described^17^ and used between passages 4 and 6^5^.

Human umbilical vein EC were isolated from umbilical cords using collagenase as previously described.^5^ Peripheral blood lymphocytes from healthy individuals were isolated by centrifugation on Histopaque 1077 (Sigma-Aldrich, Poole, UK) followed by panning on plastic.^5^ Lymphocytes were washed, counted and adjusted to a final concentration of 2×10^6^/mL in M199 supplemented with 0.15% bovine serum albumin (BSA; Sigma) and 35 µg/mL gentamycin (M199BSA).

All human samples were obtained with written, informed consent and approval from the Human Biomaterial Resource Centre (Birmingham, UK), West Midlands and Black Country Research Ethics Committee, North East Tyne and West South Research Ethics Committee, or University of Birmingham Local Ethical Review Committee in compliance with the Declaration of Helsinki.

### Lymphocyte recruitment to cocultures from flow

Endothelial-fibroblast cocultures were established on opposite sides of 0.4 µm pore Transwell filter inserts (BD Pharmingen, Cowley, UK) for 48 hours prior to treatment with or without 100 U/mL tumour necrosis factor alpha (TNFα; R&D Systems, Abingdon, UK) and 10 ng/mL interferon gamma (IFNγ; Peprotech, London, UK) for a further 24 hours as previously described.^5^ In some experiments, neutralising antibodies against IL-6 (clone 6708) or TGF-β_1_ (clone 9016; both 10 µg/mL; R&D Systems) were added alone or in combination when cocultures were established.^5 18 19^ Neutralising antibodies were present throughout the coculture and cytokine stimulation. A flow-based adhesion assay^5^ (see online supplementary methods) was used to analyse lymphocyte recruitment from flow.

10.1136/annrheumdis-2017-211286.supp1 Supplementary material 1

### Gene expression analysis

Isolated EC mRNA^5^ (RIN≥7.80) was analysed by qPCR using Taqman Universal PCR Master Mix^20^ and Assay on Demand primer kits according to manufacturer’s instructions (Applied Biosystems, Warrington, UK). Samples were analysed using 7900HT Real-Time PCR machine and SDS 2.4 (Applied Biosystems), and expressed as 2^−ΔCT^ relative to 18S.

### Flow cytometry

Expression of intracellular adhesion molecule-1 (ICAM-1) and vascular cell adhesion molecule-1 (VCAM-1) on cytokine-stimulated EC mono and cocultures were analysed by flow cytometry (see online supplementary methods). Data are expressed as median fluorescent intensity.

### Quantification of soluble mediators

Soluble agents in culture supernatants were quantified using IL-6 DuoSet ELISA, sIL-6R Quantikine ELISA Kit or VersaMAP Luminex according to manufacturer’s instructions (R&D Systems).

### Statistical analysis

Multivariant data were analysed using analysis of variance with Dunnett post-test or Kruskal-Wallis test with Dunn post-test. Alternatively, Mann-Whitney U test, Wilcoxon signed-rank test or unpaired t-test was performed. p<0.05 was considered as statistically significant.

## Results

### Demographic and baseline clinical characteristics of patients

The characteristics of the patients are shown in table 1. There was no significant difference in age, gender, 28 swollen joint counts, 28 tender joint counts, patient global visual analogue scale score, non-steroidal anti-inflammatory drug usage and ultrasound power Doppler score at the biopsied joint^14^ between clinical outcome groups. As expected, patients with RA undergoing joint replacement surgery had experienced symptoms for significantly longer than those with resolving synovitis and very early RA. However, there was no difference in symptom duration between patients with resolving synovitis or very early RA. Patients with resolving disease had significantly lower DAS28 (Disease Activity Score 28) erythrocyte sedimentation rate (ESR) at baseline, ESR and C-reactive protein when compared with patients undergoing joint replacement, but not those with very early RA. Patients with very early RA had a significantly higher ultrasound greyscale grade at the biopsied joint when compared with patients with resolving synovitis. Patients with resolving arthritis were diagnosed as having unclassified arthritis (n=6), parvovirus (n=3), reactive arthritis (n=2), pseudogout (n=1) and RA (n=2) according to established criteria. Of note, the two patients diagnosed with resolving RA had no evidence of joint-related soft tissue swelling on final examination. In both patients, synovitis resolved rapidly after briefly fulfilling criteria at presentation and no DMARDs were used in their treatment. All individuals with resolving arthritis were negative for rheumatoid factor and anticitrullinated protein antibody.

**Table 1 Demographic and baseline p characteristics:** 

	NI (n=11)	Resolving (n=14)	VeRA (n=11)	JRep (n=13)
Age (years)†	42 (34–47)	40 (32–66)	49 (48–60)	59 (39–62)
Female, n (%)	5 (45)	4 (29)	5 (45)	9 (69)
Symptom duration (weeks)†	‡	6 (4–7)	6 (4–9)	1040 (780–1098)**, *****
DAS28 ESR at baseline§	‡	3.8±1.3	4.7±1.5	5.4±1.2*
ESR (mm/hour)†	‡	9.5 (5–27)	25 (10–58)	37 (19–59)*
CRP (mg/L)†	‡	8.5 (0–14)	26 (0–45)	32 (15–56)*
RF positive (%)	‡	0 (0)	5 (45)	11 (85)**
ACPA positive (%)	‡	0 (0)***	7 (64)	**–**
SJC28†	‡	3 (2–6)	4 (3–9)	9 (4–14)
TJC28†	‡	3 (1–6)	6 (3–13)	7 (2–12)
VAS†	‡	41 (28–79)	46 (16–70)	64 (42–86)
US GS†	‡	2 (1–2)	2 (2–3)****	‡
US PD†	‡	1 (0–1)	2 (0–2)	‡
NSAID (%)	‡	9 (64)	7 (64)	8 (62)

Kruskal-Wallis test showed a significant effect of outcome group on DAS28 baseline, ESR, CRP (p<0.05), symptom duration and RF positive (p<0.001).

*p<0.05 and **p<0.01 compared with the resolving cohort by Dunn’s post-test; ***p<0.01 compared with the VeRA by Wilcoxon signed-rank test; ****p<0.01 compared with the resolving by Mann-Whitney U test; *****p<0.01 compared with the VeRA cohort by Dunn’s post-test.

†Median (IQR).

‡Data not obtained from patients at time of presentation.

§Mean±SD.

ACPA, anticitrullinated protein antibody; CRP, C-reactive protein; DAS28, Disease Activity Score 28; ESR, erythrocyte sedimentation rate; JRep, joint replacement; NI, non-inflamed; NSAID, non-steroidal anti-inflammatory drugs; RF, rheumatoid factor; SJC28, 28 swollen joint counts; TJC28, 28 tender joint counts; US GS, ultrasound greyscale grade at the biopsied joint; US PD, ultrasound power Doppler grade at the biopsied joint; VAS, visual analogue scale; VeRA, very early RA.

### Fibroblasts from VeRA lose an immunosuppressive phenotype before becoming proinflammatory

**Figure 1:**
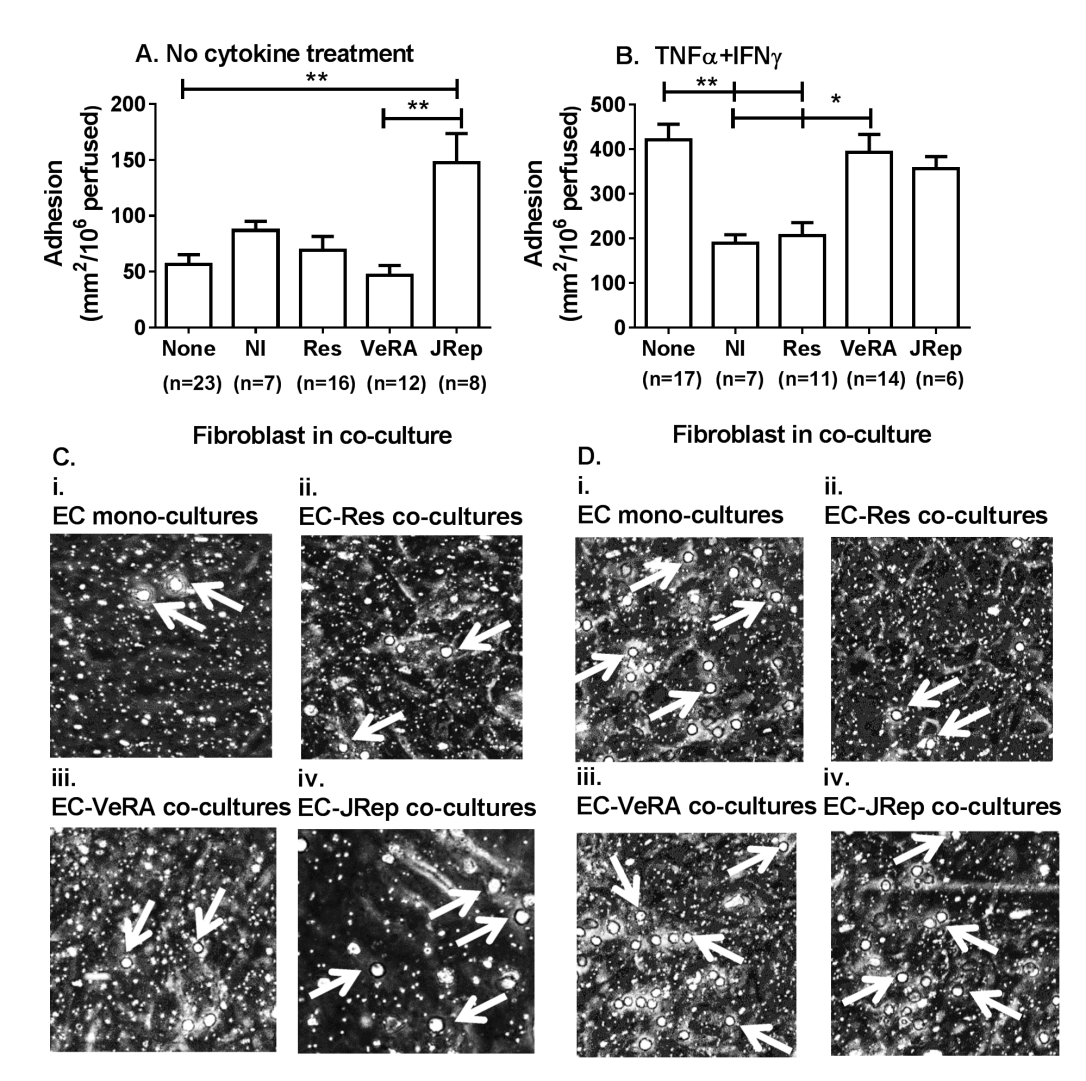
Fibroblasts from patients with resolving and persistent arthritis differentially modulate lymphocyte recruitment from flow. Cocultures were established by culturing endothelial cells and fibroblasts on opposite sides of a porous insert, prior to treatment (A) without or (B) with TNFα+IFNγ for 24 hours. Endothelial monolayers without fibroblasts (none) were used as controls. Lymphocytes were perfused and their interactions with endothelial cells were assessed by digital microscopy. (C, D) Micrograph images showing lymphocyte adhesion to (i) endothelial cells cultured alone, with fibroblasts from (ii) resolving, (iii) VeRA or (iv) JRep patients (C) in the absence of cytokine treatment and (D) in response to TNFα+IFNγ treatment. White arrow indicates an adherent lymphocyte. In A and B, Kruskal-Wallis test shows a significant effect of fibroblasts on lymphocyte adhesion (p<0.01). Data are the mean±SEM for n experiments; each incorporated a different donor for all three cell types. *p<0.05 and **p<0.01 by Dunn post-test. EC, endothelial cells; IFNγ, interferon gamma; JRep, joint replacement; NI, non-inflamed; Res, resolving; TNFα, tumour necrosis factor alpha; VeRA, very early RA.

We have previously reported that fibroblasts from joints of patients with advanced RA directly induce leucocyte recruitment in the absence of exogenous cytokines.^5 10^ In this model, fibroblasts from patients with RA undergoing joint replacement, but not very early RA, significantly increased lymphocyte adhesion when compared with untreated EC monocultures (figure 1A). Moreover, in the absence of exogenous cytokines, similar levels of binding were observed when fibroblasts from non-inflamed, resolving or very early RA tissue were incorporated into coculture (figure 1A).

Using a model of inflammation where cultures were stimulated with inflammatory cytokines, we examined the ability of synovial fibroblasts from different outcome groups to influence the cytokine-induced endothelial recruitment of lymphocytes. Fibroblasts from non-inflamed joints and resolving synovitis were immunosuppressive, inhibiting lymphocyte recruitment to TNFα+IFNγ-treated endothelium (figure 1B). By contrast, this effect was not observed when fibroblasts from patients with RA (either very early or longer duration disease) were incorporated into coculture. These fibroblasts no longer suppressed recruitment but rather supported lymphocyte adhesion at similar levels to those observed on cytokine-treated EC monocultures (figure 1B).

Collectively, these data indicate that fibroblasts from patients with very early RA are functionally distinct from both resolving synovitis and long-established disease, existing in a transitional state.

Unless otherwise stated, all future experiments were performed using cytokine-treated cocultures incorporating resolving or very early RA fibroblasts.

### Role of IL-6 and TGF-β_1_ in effects of resolving and very early RA fibroblasts in coculture

**Figure 2:**
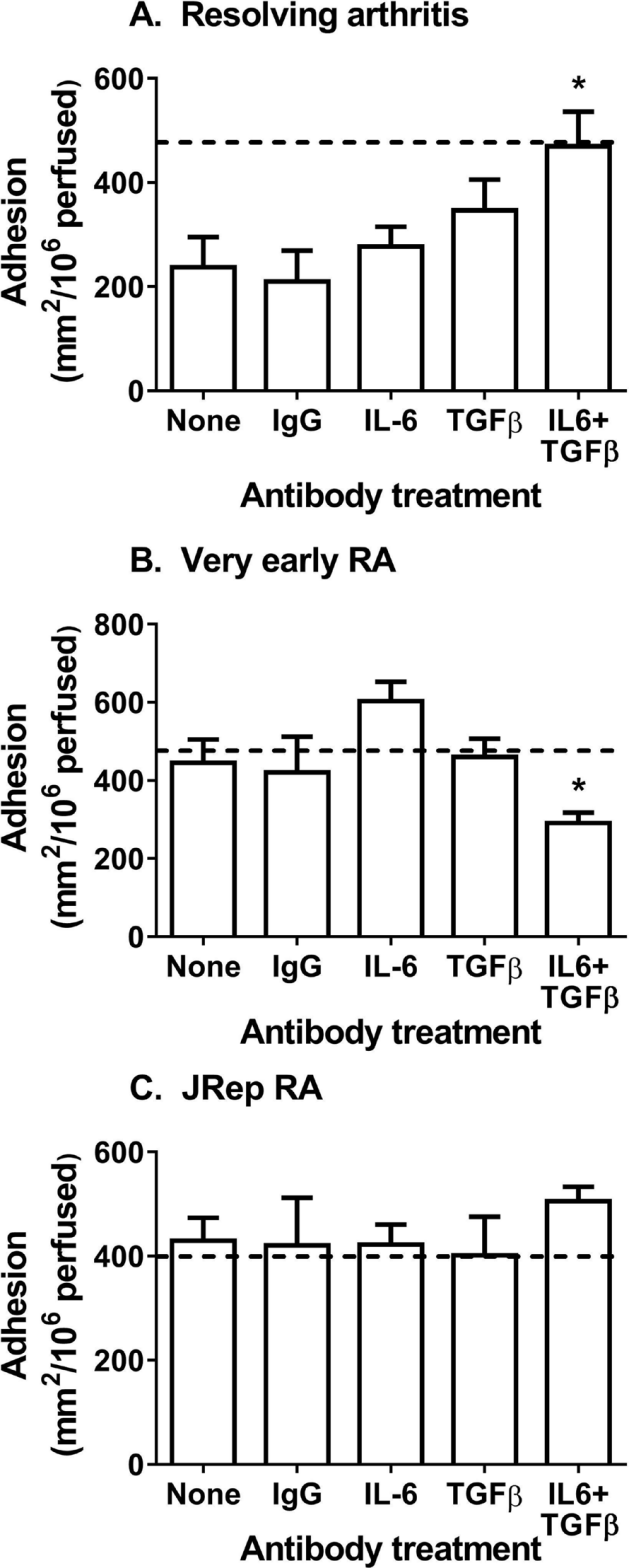
Resolving fibroblasts mediated immunosuppressive effect through IL-6 and TGF-β_1_. Actions of IL-6 or TGF-β_1_ were neutralised, alone or in combination, in TNFα+IFNγ-treated cocultures incorporating fibroblasts from patients with (A) resolving synovitis, (B) very early RA or (C) joint replacement RA (JRep). Dotted line (-----) represents adhesion to TNFα+IFNγ-treated endothelial monocultures for paired experiments. IgG represents cocultures incubated with isotype control antibodies. In A and B, ANOVA shows a significant effect of antibody treatment on lymphocyte adhesion (p<0.01). Data are the mean±SEM from three to five independent experiments each incorporating a different donor for all cell types. *p<0.05 compared with None (untreated cocultures) by Dunnett post-test. ANOVA, analysis of variance; IFNγ, interferon gamma; IL-6, interleukin 6; JRep, joint replacement; RA, rheumatoid arthritis; TGF-β, transforming growth factor beta; TNFα, tumour necrosis factor alpha.

The immunosuppressive response of mesenchymal stromal cells from healthy tissues is facilitated by common bioactive mediators, IL-6 and TGF-β_1_.^5 18 19^ It is possible that such endogenous pathways are corrupted early in the pathogenesis of RA. Neutralisation of both IL-6 and TGF-β_1_ significantly blocked the inhibitory effects of resolving fibroblasts in coculture (figure 2A). In contrast, neutralisation of IL-6 and TGF-β_1_ in very early RA cocultures significantly reduced lymphocyte adhesion (figure 2B), restoring immunoprotective functions to those of resolving cocultures. Interestingly, blockade of IL-6 and TGF-β_1_ had no effect on lymphocyte recruitment to cocultures incorporating RA fibroblasts from joint replacement patients (figure 2C). In all conditions, single antibody blockade or the presence of isotype antibodies had no effect on adhesion (figure 2A–C). Thus, IL-6 and TGF-β_1_ had essentially opposite effects on cytokine-treated cocultures with either resolving or very early RA fibroblasts.

**Figure 3:**
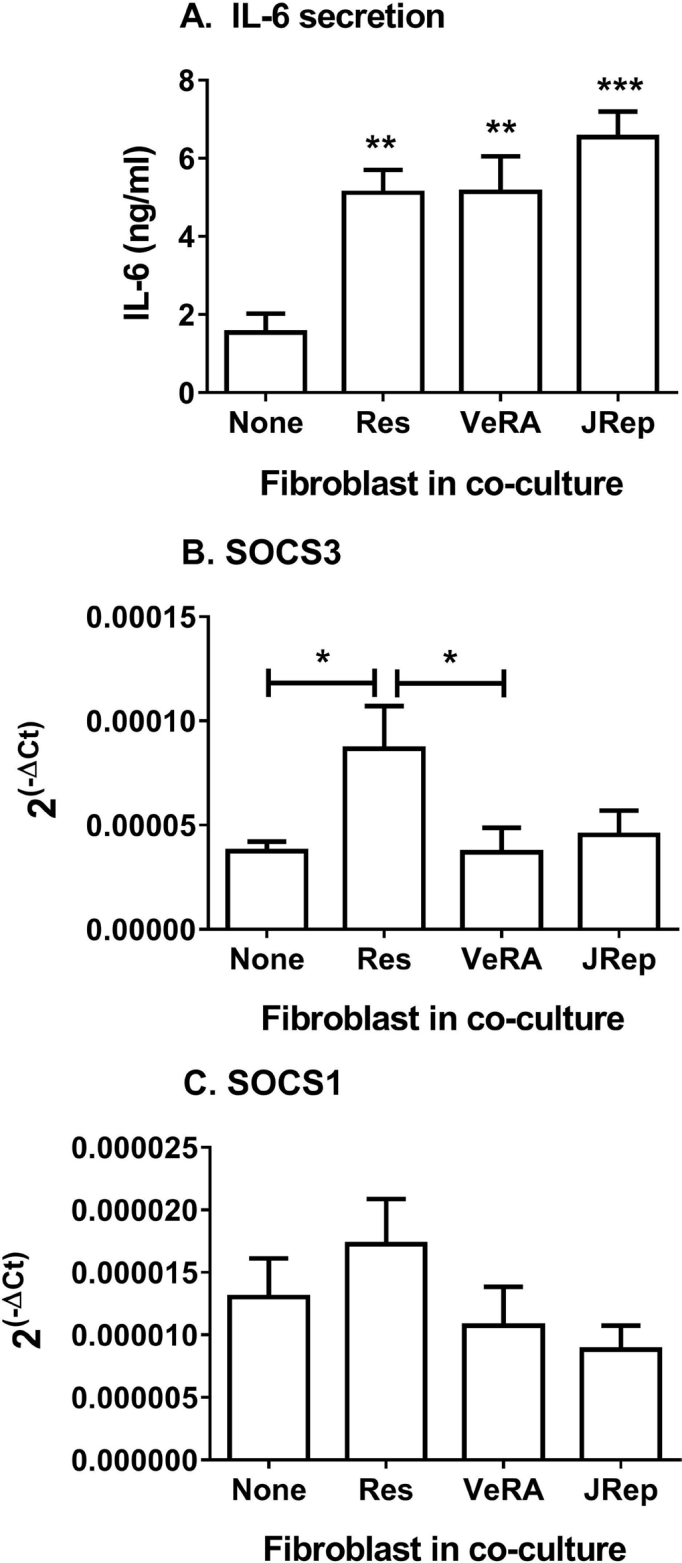
Secretion and signalling of IL-6 in cocultures. (A) IL-6 release during TNFα+IFNγ-treated cocultures. ANOVA shows a significant effect of culture conditions on the secretion of IL-6 (p<0.001). (B) SOCS3 and (C) SOCS1 gene expression analysed by qPCR. Data are expressed as 2^−ΔCT^ relative to 18S expression. Data are the mean±SEM from three to five independent experiments each incorporating a different donor for all cell types. *p<0.05, **p<0.01 and ***p<0.001 compared with None (endothelial monoculture) by Dunnett post-test, unless otherwise indicated. ANOVA, analysis of variance; IFNγ, interferon gamma; IL-6, interleukin 6;  JRep, joint replacement; Res, resolving; SOCS, suppressor of cytokine signalling; TNFα, tumour necrosis factor alpha; VeRA, very early RA.

Exploring this further, we detected significantly more IL-6 in supernatants from cocultures compared with endothelial monocultures following cytokine treatment (figure 3A). However, there was no difference between the clinical outcome groups (figure 3A). Of note, resting fibroblasts from different disease stages release comparable levels of IL-6 in culture (online supplementary figure 1A). Soluble IL-6R transcripts in EC were also similar between all culture conditions tested (online supplementary figure 1B); however, we were unable to detect measurable levels of sIL-6R released by these cultures. Suppressor of cytokine signalling 3 (SOCS3) and SOCS1 regulate signal transducer and activator of transcription (STAT) activation in response to IL-6.^21^ Here, expression of SOCS3 was upregulated in EC from resolving cocultures, but not in EC from very early RA cocultures (figure 3B). In contrast, SOCS1 expression in EC remained unchanged upon coculture (figure 3C). Downstream signalling from IL-6 differed between the two forms of cytokine-activated cocultures.

### Profile of secretome released by resolving and VeRA fibroblasts in coculture

**Figure 4:**
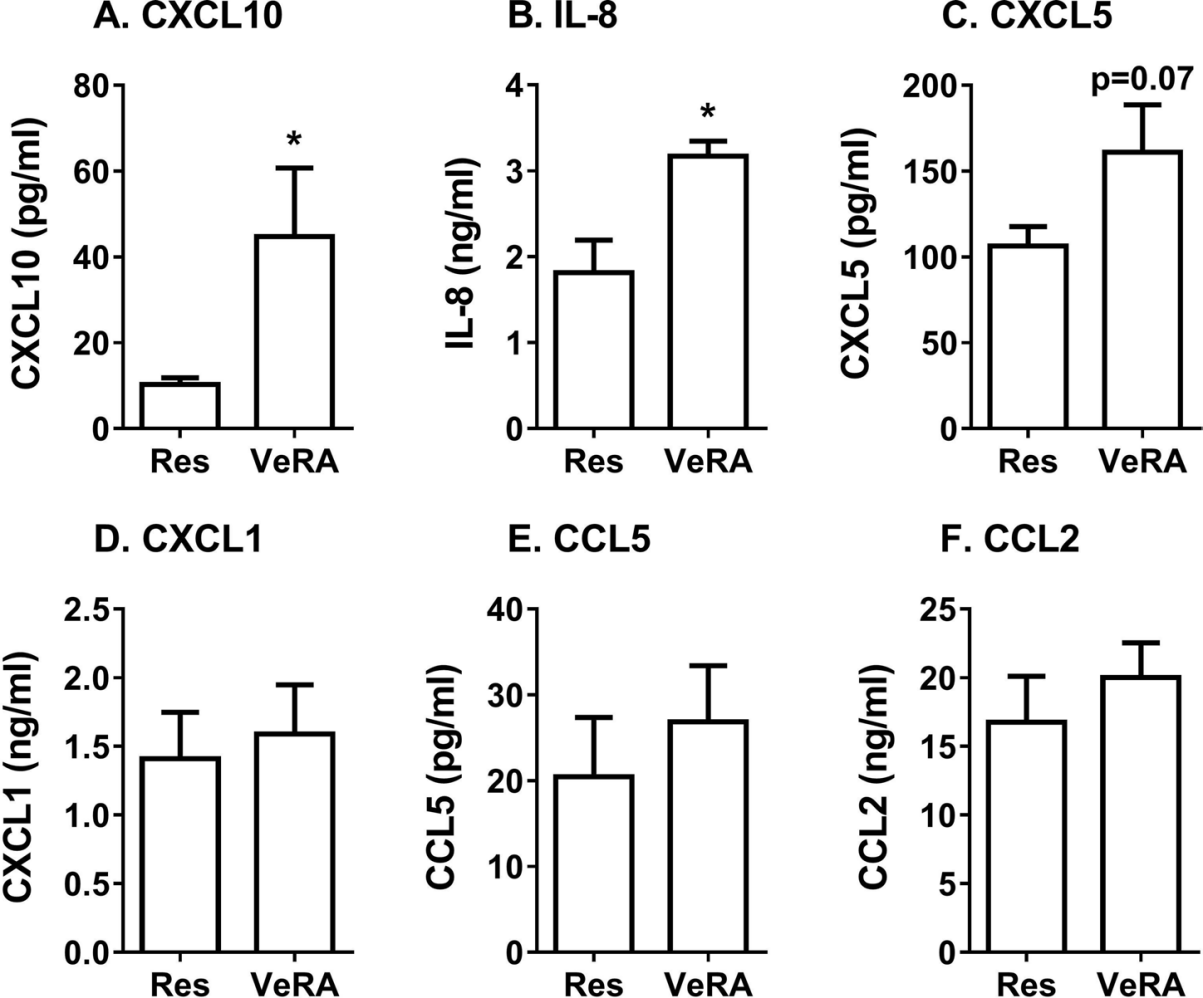
Secretome from resolving and very early RA cocultures. Conditioned media from resolving or very early RA fibroblast cocultures were measured by multiplex analysis. (A) CXCL10, (B) IL-8, (C) CXCL5, (D) CXCL1, (E) CCL5 and (F) CCL2 expression. Data are the mean±SEM from five to nine independent experiments each incorporating a different donor for all cell types. *p<0.05 by unpaired t-test. IL-8, interleukin 8, also known as CXCL8; Res, resolving; VeRA, very early RA.

We also wondered whether very early RA fibroblasts altered the secretome generated during coculture, such that it was no longer immunosuppressive. Using multiplex analysis, we detected significantly higher levels of the chemokines CXCL10 and IL-8, and a tendency for higher CXCL5 in the very early RA coculture supernatants when compared with the resolving cocultures (figure 4A–C). However, expression of the chemokines CXCL1, CCL5 and CCL2 was comparable between both coculture conditions (figure 4D–F), while IL-4, IL-10 and IL-1α were undetectable. We observed no significant difference in the concentration of these chemokines released by resolving and very early RA monocultures, although overall levels were lower than that found in cocultures (online supplementary figure 2). Therefore, the composition, and potentially the bioactivity, of the secretome appeared to differ between resolving and very early RA cocultures.

10.1136/annrheumdis-2017-211286.supp2 Supplementary Figure 1

### Analysis of gene expression in EC upon coculture

To further investigate the loss of suppression of lymphocyte adhesion, we analysed the expression of adhesion molecules and chemokines by inflamed EC upon coculture. Comparing EC from resolving and very early RA cocultures, we detected no difference in the expression of ICAM-1 or VCAM-1 (online supplementary figure 3), or CXCR3 ligand transcripts (data not shown). While coculture significantly reduced expression of E-selectin mRNA compared with cytokine-treated EC alone (data not shown), this effect was similar for each disease outcome. Thus, changes in the expression of the genes analysed showed no clear differences between disease outcomes or correlation with the functional differences in recruitment observed with resolving and very early RA cocultures.

10.1136/annrheumdis-2017-211286.supp3 Supplementary Figure 2

## Discussion

Little is known about how the development of RA alters the immunomodulatory properties of synovial fibroblasts. We examined for the first time the effects of phase of disease and disease outcome on synovial fibroblast regulation of the inflammatory infiltrate through crosstalk with EC. Synovial fibroblasts show outcome-specific and stage-specific effects. Upon coculture with EC, fibroblasts from resolving synovitis suppressed lymphocyte adhesion in response to cytokines. This immunoprotective effect was lost in fibroblasts from very early RA, allowing increased lymphocyte recruitment. Hence, fibroblasts cultured from tissues with divergent disease outcomes (resolving vs persistence) are functionally distinct. Moreover, fibroblast–EC interactions evolve with RA progression. In contrast to established disease, fibroblasts from very early RA have not yet acquired the ability to autonomously activate EC in the absence of exogenous cytokines. Thus, we have shown for the first time that synovial fibroblasts undergo two distinct functional changes as RA evolves: first the early loss of immunosuppressive capability, and second the slower acquisition of an intrinsically stimulatory phenotype during disease progression.

IL-6 and TGF-β are pleiotropic cytokines, each able to induce divergent proinflammatory or anti-inflammatory effects depending on the inflammatory context or cell type (reviewed by ref 1). Moreover, emerging evidence reveals complex and intricate crosstalk between IL-6 and TGF-β_1_ signalling pathways, in which each cytokine can positively^22–24^ or negatively^25–27^ regulate the expression or activity of the other depending on the inflammatory context. Using T cell biology as an example, TGF-β_1_ inhibited the production of the IL-6 inhibitor SOCS3, thus prolonging IL-6 signalling to initiate Th17 differentiation.^28^ Conversely, IL-6 augmented expression of the TGF-β signalling inhibitor SMAD7, preventing TGF-β_1_-induced T_Reg_ differentiation.^26^ In the context of leucocyte recruitment, treatment with recombinant IL-6 or TGF-β_1_ or both suppressed neutrophil infiltration into lipopolysaccharide (LPS)-inflamed lungs.^29^ Such apparently divergent, contextually determined roles are seen in our study. Here, IL-6 and TGF-β_1_ were identified as the bioactive agents required for the inhibitory effects on recruitment of cocultured resolving fibroblasts. Similar findings have been reported for stromal cells from non-inflamed tissues,^5 18^ suggesting the existence of shared stromal immunoprotective mechanisms. In contrast, neutralisation of IL-6 and TGF-β_1_ inhibited the prorecruitment effect of cocultured very early RA fibroblasts. This suggests that in VeRA, IL-6 and TGF-β_1_ have not simply lost efficacy, but trigger stimulatory rather than inhibitory downstream events.

Synovial fibroblasts are a major source of IL-6 in RA,^30^ which we also observed in our EC-fibroblast cocultures. IL-6 can signal through its membrane-bound (CD126; IL-6R) or soluble receptor (sIL-6R) (reviewed by ref 31). Indeed, synovial fibroblasts induce STAT3 phosphorylation and activation in response to sIL-6R engagement.^32–34^ The absence of detectable sIL-6R in our supernatants (both measured here and previously^10^) strongly indicates that IL-6 released during coculture signals through CD126 expressed by EC,^5^ but not fibroblasts. Given that fibroblasts cannot respond to IL-6 generated during coculture, distinct fibroblast–EC interactions must regulate EC responses to IL-6 and produce the discrete patterns of lymphocyte recruitment that we observed. Here, we observed two different patterns of expression of the negative regulator, SOCS3, in EC from resolving and very early RA cocultures. We hypothesise that high SOCS3 expression (ie, negative regulation of STAT activation), as seen in the EC from resolving cocultures, triggers an immunoprotective IL-6 response. Conversely, failure to induce SOCS3 was associated with loss of immunosuppressive responses in the EC from very early RA cocultures. Such a situation has been observed in adjuvant-induced arthritis, where low endothelial SOCS3 levels, and therefore negative regulation of IL-6 signalling, has been linked with more severe arthritis and elevated neutrophil influx into the joint.^35^ Collectively, these data reveal two distinct IL-6 signalling pathways in EC from cocultures, which are induced in a disease outcome-specific manner and elicit different functional consequences in EC.

Our data clearly show that IL-6 acts synergistically with TGF-β_1_ to mediate the differential effects on lymphocyte adhesion to inflamed EC in coculture. TGF-β_1_ is secreted in its bioactive form by a variety of cell types, including EC and fibroblasts. However, the ELISA kits available during this study only measured total TGF-β_1_ after acid activation, rather than bioactive TGF-β_1_. Therefore, it is not possible to distinguish which cell type was secreting bioactive TGF-β_1_. EC in all conditions expressed similar transcript levels of the three TGF-β receptors (data not shown), indicating that EC were potentially capable of responding to TGF-β_1_ produced during coculture. The requirement to understand the inflammatory context of TGF-β_1_ production is once again emphasised by conflicting findings on the impact (suppressive^36^ vs stimulatory^37^) of TGF-β_1 _on in vivo models of arthritis, where exogenous TGF-β_1_ treatment either exacerbated,^38 39^ alleviated^40^ or had no effect^41^ on disease severity.

Biological therapies that target IL-6 and its receptor (eg, tocilizumab) are efficacious in RA including for those individuals who do not respond to anti-TNFα treatment.^42 43^ Although we detected similar concentrations of IL-6 in cocultures, we did observe a different profile of soluble mediators released by resolving and very early RA fibroblast cocultures. This raises the intriguing possibility that the bioactivity of the secretome is different between the two cocultures, where soluble agents exclusive to the very early RA cocultures alter IL-6 and TGF-β_1_ responses to generate a stimulatory effect. Moreover, difference in the secretomes by resolving and very early RA cocultures could influence the presentation of chemokines by the endothelium and therefore might account for the altered lymphocyte adhesion profiles observed here. For example, fibroblast-induced production of proteases during coculture can adversely affect lymphocyte binding.^44^ Further work is required to identify the exact soluble mediator(s) responsible for these changes, as they are likely to offer novel targets for early therapeutic intervention in RA.

Failure to suppress recruitment may represent a manifestation of the transition the stroma undergoes as the disease progresses. It is unlikely that such changes in very early RA are due to the fibroblasts passively responding to local inflammatory responses. Instead, fibroblasts with a transitional functional phenotype will actively contribute to disease development and persistence, further fuelling the evolution of their phenotype towards so-called ‘imprinted aggressors’ (eg, ref 45). Emerging evidence strongly indicates such reprogramming is due to accumulated epigenetic modifications,^46 47^ which may directly alter the production of proinflammatory mediators or modify the balance of microRNAs (eg, mir155, 146, 203) within the fibroblast.^(^e.g, ref 48^)^ This could explain how the regulation of IL-6 mRNA stability becomes altered in rheumatoid synovial fibroblasts, where the negative regulator Zc3h12a (RNA-binding protein) switches its activity to positively stabilise IL-6 mRNA.^49^ Epigenetic modifications in the earliest phases of disease could also account for the differential effects seen in this study by resolving and VeRA fibroblasts in coculture.

Our study is not the first to indicate that Vvery early RA is subtly different from resolving arthritis or established RA. Patients with very early disease have a distinct serum metabolomic profile and synovial fluid cytokine profile when compared with patients with established RA.^15 50^ Moreover, fibroblasts from very early RA showed increased dickkopf-related protein 1 (DKK-1) expression with the potential to adversely alter bone remodelling; a feature not apparent in fibroblasts from patients with resolving synovitis.^51^ Collectively, these data strongly support the transitional nature of synovial pathology during the earliest stages of disease development. Early interventions targeting ‘pathogenic’ fibroblasts may therefore be required in order to restore protective regulatory processes.

10.1136/annrheumdis-2017-211286.supp4 Supplementary Figure 3
