# Supplementary material for: Identification of a transitional fibroblast function in very early rheumatoid arthritis
Source: Ann Rheum Dis. 2017 Aug 28;76(12):2105–12. doi: 10.1136/annrheumdis-2017-211286 (PMC5705853; doi:10.1136/annrheumdis-2017-211286)
Supplement: Supplementary material 1 [file annrheumdis-2017-211286supp001.docx]

**Supplementary Methods**

**Materials and Methods**

*Lymphocyte recruitment to co-cultures from flow*

Fibroblasts (5x10^5^) were seeded onto inverted 0.4µm pore Transwell filter inserts (BD Pharmingen, Cowley, UK) as previously described^6^ and cultured for 24h. EC were then seeded on the inner surface of inserts. Cells were co-cultured for 48h prior to treatment with or without 100U/ml TNFα (R&D Systems, Abingdon, UK) and 10ng/ml IFNγ (Peprotech Inc., London, UK) for a further 24h. Filters were incorporated into a parallel-plate flow chamber and attached to a perfusion system mounted on the stage of a phase-contrast digital microscope, all at 37^o^C, as previously described^6^. Lymphocytes were perfused over endothelium for 4min at 0.1Pa. Digitised recordings were made after 2min of washout and analysed offline using Image-Pro Plus software (Media Cybernetics UK, Marlow, UK). Lymphocytes bound to the endothelium were counted, and the total number of adherent cells averaged per field and expressed as adherent lymphocytes/mm^2^/10^6^ cells perfused^6^.

*Flow Cytometry of endothelial surface receptors*

EC mono- or co-cultures were incubated with accutase (Gibco) for 5min. Cells were retrieved, washed, and incubated with FITC-conjugated antibody against CD106 (VCAM-1) or APC-conjugated antibody against CD54 (ICAM-1) (clones 51-10C9 and A58 respectively; BD Biosciences, UK) for 30min at 4^o^C. FITC-conjugated and APC-conjugated mouse IgG1 (from BD Biosciences and eBiosciences respectively) were used as the negative controls. All samples were washed and analysed using a Dako Cyan flow cytometer. Data was expressed as median fluorescent intensity (MFI) minus the IgG.
