# Supplementary material for: Identification of a transitional fibroblast function in very early rheumatoid arthritis
Source: Ann Rheum Dis. 2017 Aug 28;76(12):2105–12. doi: 10.1136/annrheumdis-2017-211286 (PMC5705853; doi:10.1136/annrheumdis-2017-211286)
Supplement: Supplementary Figure 1 [file annrheumdis-2017-211286supp002.docx]

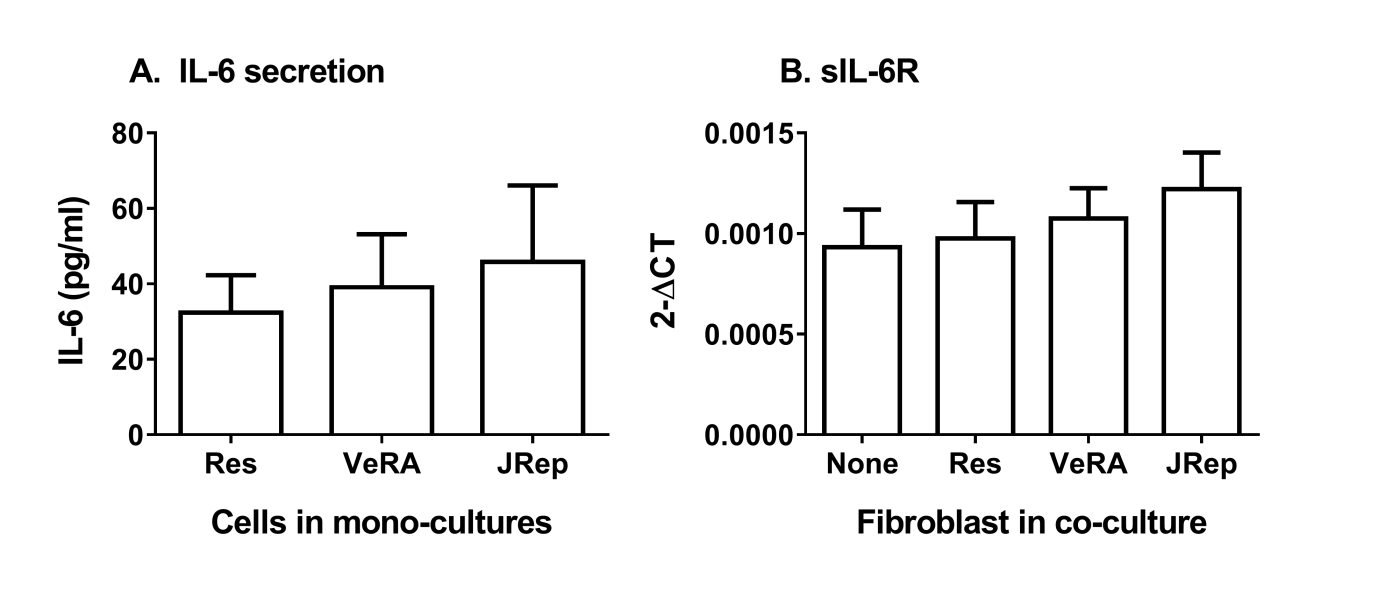


**Supplementary Figure 1: IL-6 secretion and downstream induction of gene expression.**

**(A)** IL-6 release from resting fibroblast mono-cultures. **(B)** Soluble IL-6 receptor transcript expression in endothelial cells from mono-cultures or co-cultures by qPCR. Data are expressed as 2^-ΔCT^ relative to 18S expression. Data are the mean ± SEM from 3-5 independent experiments each incorporating a different donor for all cell types.
