# Supplementary material for: Identification of a transitional fibroblast function in very early rheumatoid arthritis
Source: Ann Rheum Dis. 2017 Aug 28;76(12):2105–12. doi: 10.1136/annrheumdis-2017-211286 (PMC5705853; doi:10.1136/annrheumdis-2017-211286)
Supplement: Supplementary Figure 2 [file annrheumdis-2017-211286supp003.docx]

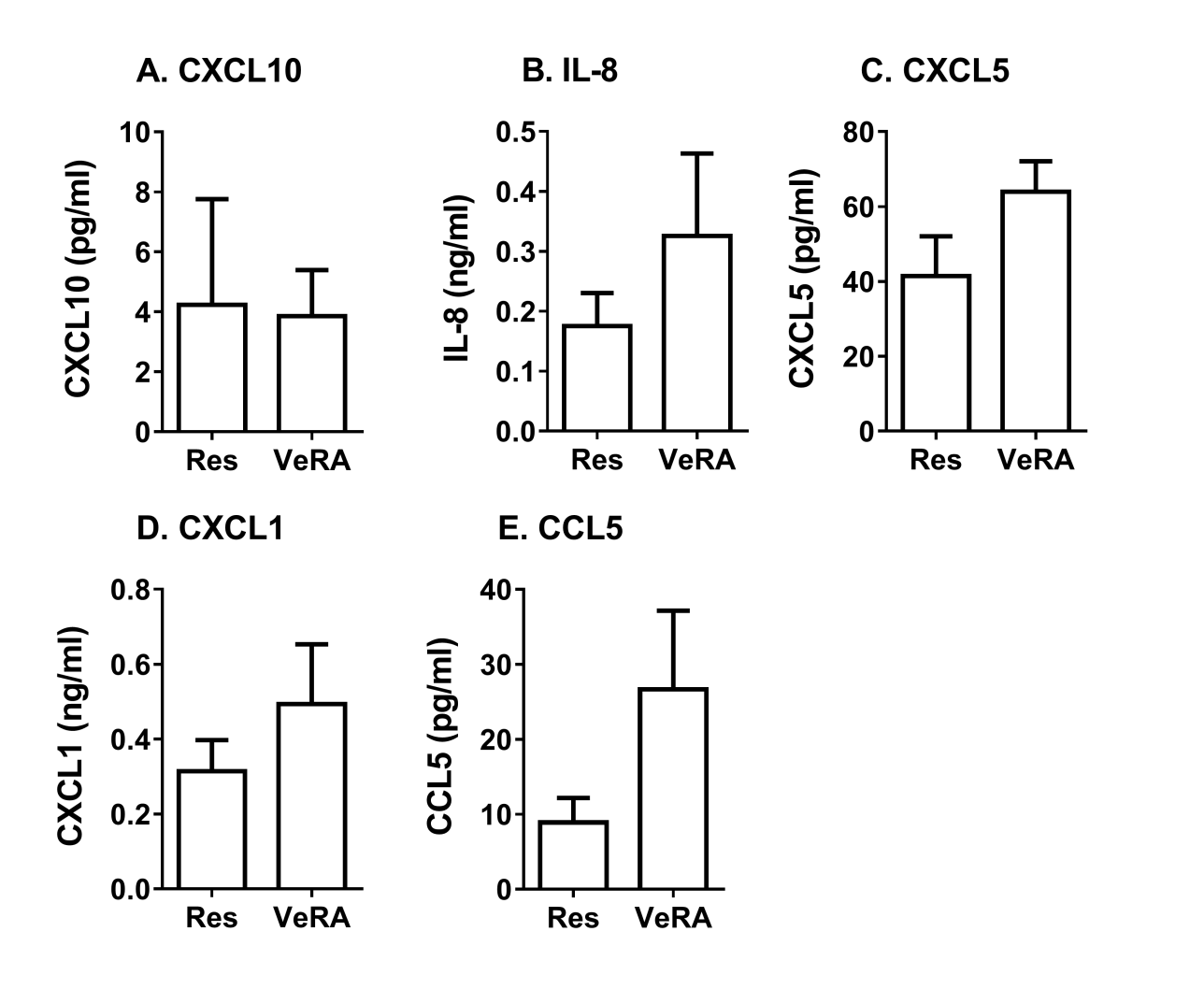


**Supplementary Figure 2: Secretome from resolving and very early RA mono-cultures.**

Conditioned media from resolving or very early RA fibroblast mono-cultures were measured by multiplex analysis. **(A)** CXCL10, **(B)** IL-8, **(C)** CXCL5, **(D)** CXCL1 and **(E)** CCL5 expression. Data are the mean ± SEM from 3-7 independent experiments each incorporating a different donor for all cell types.
