# Supplementary material for: Identification of a transitional fibroblast function in very early rheumatoid arthritis
Source: Ann Rheum Dis. 2017 Aug 28;76(12):2105–12. doi: 10.1136/annrheumdis-2017-211286 (PMC5705853; doi:10.1136/annrheumdis-2017-211286)
Supplement: Supplementary Figure 3 [file annrheumdis-2017-211286supp004.docx]

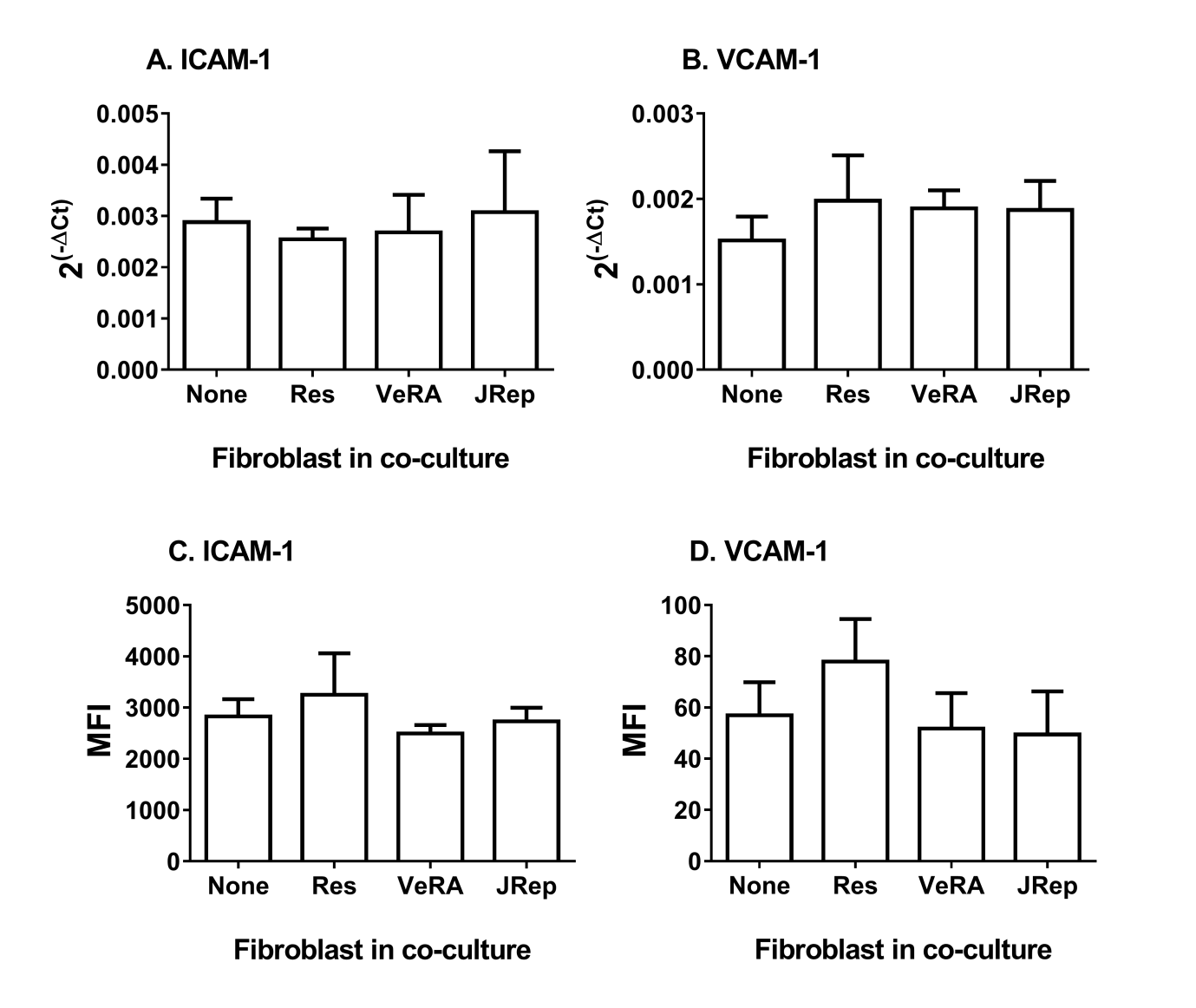


**Supplementary Figure 3: Expression of adhesion molecules in endothelial cells.**

**(A)** ICAM-1 and **(B)** VCAM-1 transcript expression in endothelial cells from mono-cultures or co-cultures by qPCR. Data are expressed as 2^-ΔCT^ relative to 18S expression. **(C)** ICAM-1 and **(D)** VCAM-1 surface protein expression on endothelial cells from mono-cultures or co-cultures by flow cytometry. Data are expressed as MFI minus IgG control. Data are the mean ± SEM from 3-6 independent experiments each incorporating a different donor for all cell types.
